# Self-diploidization of human haploid parthenogenetic embryos through the Rho pathway regulates endomitosis and failed cytokinesis

**DOI:** 10.1038/s41598-017-04602-y

**Published:** 2017-06-26

**Authors:** Lizhi Leng, Qi Ouyang, Xiangyi Kong, Fei Gong, Changfu Lu, Lei Zhao, Yun Shi, Dehua Cheng, Liang Hu, Guangxiu Lu, Ge Lin

**Affiliations:** 1 Institute of Reproductive & Stem Cell Engineering, Central South University, Changsha, 410078 China; 2 Key Laboratory of Stem Cells and Reproductive Engineering, Ministry of Health, Changsha, 410078 China; 3 National Engineering and Research Center of Human Stem Cell, Changsha, 410078 China

## Abstract

A diploid genome is necessary for normal mammalian development, thus haploid parthenogenetic embryos undergo frequent self-diploidization during preimplantation development; however, the underlying mechanism is unclear. In this study, time-lapse recording revealed that human haploid parthenotes (HPs) undergo self-diploidization via failed cytokinesis (FC) and endomitosis (EM). The frequencies of FC/EM were significantly higher in HPs than in normal fertilized embryos (26.3% *vs*. 1.6%, P < 0.01; 19.7% *vs*. 0, P < 0.01), and above 90% of FC/EM occurred at the first cell cycle in HPs. Fluorescent *in situ* hybridization of chromosome 16,18 and X in HPs identified diploid recovery after the appearance of FC/EM, and FC/EM HPs showed improved blastocyst formation compared with non-FC/EM HPs (18.8% and 40.0% *vs*. 15.4%, P > 0.05). In 66.7% of the 1-cell stage HPs, furrow ingression was not observed during the time for normal cleavage, and both immunostaining and gene expression analysis of 1-cell stage HPs revealed the absence or down-regulation of several key genes of the Rho pathway, which regulates cytomitosis. Our results suggested that the major mechanism for self-diploidization is Rho pathway inhibition leading to FC/EM in the first cell cycle, and fine-tuning of this signalling pathway may help to generate stable haploid embryos for stem cell biology studies.

## Introduction

Haploid development is a normal part of the life cycle in some lower animals^1^, but it is not observed in mammals. Studies in mice have revealed that the preimplantation developmental potential of haploids is significantly impaired relative to diploid embryos^2–6^. Mammalian haploid parthenotes (HPs) are able to undergo several cycles of cell division after oocyte activation, but never proceed to term, arresting at different stages of development^7, 8^.

A previous study showed that mouse oocytes, after artificial activation followed by second polar body extrusion, exhibited a single pronucleus and were haploid, and about 15–18% of diploid cells were identified in HPs at the later morula stage^9^. Karyotype analysis showed that all HPs exhibited mixed ploidy, including haploid and diploid cells at the blastocyst stage^10, 11^, indicating that some haploid cells had already undergone self-diploidization before the blastocyst stage. Recently, advances in culture conditions and flow cytometric cell sorting have facilitated the derivation of embryonic stem cells (ESCs) from mammalian haploid parthenogenetic and androgenetic embryos^12–15^. Nevertheless, these haploid ESCs exhibit an intrinsic tendency for diploidization following passaging *in vitro* ^13^. For this reason, long-term culture of these ESCs requires enrichment of haploid cells every 3 to 5 passages via sorting. The above data indicated that haploid cells are not stable and diploidization greatly hinders efforts to determine the gene function in numerous biological processes by mutagenesis-based genetic approaches. Although previous studies suggested that haploid cells do not become diploid via cell fusion^12, 13^, it remains unclear why and how haploid cells actively undergo diploidization during development.

In the study of polyploidization using model animals, diploid cells could become polyploid through endoreplication, including endomitosis and endocycling^16–18^. In endoreplication, a cell cycle variation generates a polyploid genome by repeated rounds of DNA replication in the absence of cell division^18^. Recently, time-lapse technology has been used to record and analyse the cleavage behaviours and developmental kinetics of human embryos through continuous monitoring^19–21^. A number of abnormal behaviours, such as non-synchronized cell cycle dynamics, direct cleavage and reverse cleavage (RC, referring to either blastomere fusion or failed cytokinesis), were associated with compromised developmental potential^22–24^. Under time lapse monitoring, early endomitotic megakaryocytes exhibited a late failure of cytokinesis, accompanied by a backward movement of the daughter cells^25, 26^. Thus, time-lapse technology can help to reveal cleavage behaviours that cannot be observed in conventional culture. Most importantly, using time-lapse images, certain cleavage behaviours that correlate with the self-diploidization of human HPs during the preimplantation period could be discovered.

In this study, we investigated whether human HPs undergo self-diploidization through specific cleavage behaviours and at which stage of development haploid cells become diploid. Furthermore, we explored the underlying mechanism that regulates self-diploidization of human HPs during preimplantation development.

## Materials and Methods

### Informed consent for oocyte donation

This study was approved and guided by the ethical committee of the Reproductive & Genetic Hospital of CITIC-XIANGYA (LL-SC-SG-2012-003 and LL-SC-SG-2012-004). All the immature oocytes came from intracytoplasmic sperm injection (ICSI) cycles at the Reproductive & Genetic Hospital of CITIC-XIANGYA and were obtained with written informed consent signed by the donor couples. The informed consent confirmed that the couple donors were voluntarily donating oocytes for research on the self-diploidization of human haploid parthenogenetic embryos with no financial payment.

### Collection of oocytes and *in vitro* maturation (IVM)

Oocytes lacking both a polar body and a germinal vesicle were collected. Immature oocytes were cultured at 37.5 °C, in an atmosphere of 6% CO_2_, 5% O_2_, and 89% N_2_. The medium used was commercial IVM medium (Vitrolife, Göteborg, Sweden) supplemented with 0.075 IU/mLFSH, 0.5IU/ml hCG, 1 μg/mL estradiol and 0.5% human serum albumin (HSA). Only those oocytes that expelled the first polar body within 4–8 hours after *in vitro* culture were collected and prepared for further experiments.

### Production of parthenogenetic embryos and *in vitro* fertilized embryos

For parthenogenetic embryos, HPs and diploid parthenotes (DPs) were prepared from *in vitro* matured oocytes by activating them for 5 min in G-MOPS containing 10 μM calcium ionophore A23187 (Sigma, Pittsburgh, USA). Oocytes were rinsed several times with G-MOPS medium, and placed in G-1 Plus media (Vitrolife) containing 10 μg/mL puromycin (Sigma) or 2 mM 6-dimethylaminopurine (6-DMAP, Sigma) for 4 h, thoroughly washed in G-MOPS medium, and finally cultured in G-1 Plus media at 37.5 °C, 6% CO_2_, 5% O_2_, and 89% N_2_, in a humidified atmosphere. After 12 h of incubation, the oocytes were assessed for extrusion of the second polar body (PB) and number of pronuclei. Normal fertilized embryos (NFEs) were obtained from *in vitro* matured oocytes by conventional ICSI.

### Embryo culture and time-lapse recording

HPs, DPs and NFEs at the pronuclear stage were moved to the wells of the pre-equilibrated EmbryoSlide (Vitrolife) and cultured in G-1 Plus media. Care was taken to remove any bubbles before placing the embryos in the wells. Slides containing embryos were placed into the Embryoscope chamber immediately and cultured in a 6% CO_2_, 5% O_2_, and 89% N_2_ atmosphere at 37.5 °C. Culture medium was changed on day 3. When the slide was removed from the Embryoscope chamber, all embryos were transferred to the same position of another pre-equilibrated EmbryoSlide containing G2 Plus medium. The slide was then returned to the Embryoscope chamber and time-lapse monitoring was continued. Images of each embryo were recorded every 10 min. Normal and abnormal division behaviours in the three initial cleavages were annotated and analysed as described previously^20^.

### Fluorescence *in situ* hybridization (FISH) analysis of HPs

The FISH procedure was performed as described previously^27^. Briefly, zona-free HPs were exposed to a hypotonic solution (1% sodium citrate in 6 mg/mL bovine serum albumin) for 5 min and transferred into Tween 20 fixative buffer (0.01 N HCl, 0.1% Tween 20; Sigma) on amine-coated slides. After isolation of nuclei, slides were air-dried and rinsed in phosphate-buffered saline (PBS) for 5 min. For hybridization, we used a DNA centromere probe panel (Vysis, Abbott Molecular Inc., Des Plaines, USA) for chromosomes 16, 18, and X. The slides were warmed to 37 °C, and then the probe mixture was added to each slide under a coverslip. Probes and nuclear DNA were denatured simultaneously at 75 °C for 5 min, and then hybridization for at least 4 h at 37 °C in a moist chamber. The slides were then washed with 0.4 × standard saline citrate (SSC)/0.3% NP-40 (Sigma) at 73 °C for 2 min and 2 × SSC/0.1% NP-40 at room temperature for 1 min. After rinsing in PBS, the slides were air-dried and mounted with 4, 6-diamidino-2-phenylindole (DAPI) (Sigma) to counterstain the nuclei. The FISH signals were observed using an Olympus BX-51 fluorescence microscope.

### Immunocytochemistry

Alkaline phosphatase activity was detected using a BCIP/NBT kit (Invitrogen, Carlsbad, CA, USA), according to the manufacturer’s protocol. For immunofluorescence staining, HPs, DPs and NFEs were collected at the first division stage. The zona pellucida was removed by a brief incubation with acidic Tyrode’s solution. Zona-free embryos or ESCs were fixed in microtubule stabilizing buffer (to stain α-Tubulin, F-actin and p-MRLC: 0.1 M PIPES, PH 6.9, 2 mM MgCl_2_.6H_2_O, 2.5 mM EGTA, 2% formaldehyde, 0.5% Triton X-100 and 10μM taxol), ice-cold 10% trichloroacetic acid (TCA, to stain RhoA), or 4% paraformaldehyde (PFA, to stain β-tubulin, SMA, AFP, OCT-4, NANOG and TRA-1-60) for 30 min. Immunostaining was performed according to standard procedures^28^ and the following primary antibodies were used: mouse anti-RhoA (1:100; Santa Cruz Biotechnology, California, USA), mouse anti-α-Tubulin (1:200, Sigma), mouse antibodies recognizing the serine 19-phosphorylated form of myosin regulatory light chain (p-MRLC, 1:200; Cell Signaling Technology, Boston, USA), BODIPY-FL phallacidin (F-actin, 1:800, Sigma), mouse anti-OCT4 (1:50; Santa Cruz Biotechnology), rabbit anti-NANOG (1:50; Abcam, Cambridge, UK), mouse anti-TRA-1-60 (1:50; Millipore, Boston, MA, USA), mouse anti-β tubulin (1:800; Sigma), mouse anti-AFP (1:500; sigma), and mouse anti-SMA (1:100; Chemicon, California, USA). Appropriate secondary antibodies were used that were conjugated with Alexa 488 or Alexa 594 (1:800; Invitrogen). DAPI (1μg/mL, Invitrogen) was used to stain the nuclei.

### Quantitative and semi-quantitative real-time reverse transcription polymerase chain reaction (RT-PCR) analysis

HPs, DPs, and NFEs were picked at the pronucleus stage and the zona pellucida was removed using acidic Tyrode’s solution. Embryos were dispensed in lysis buffer, and cDNA libraries were generated using Smart-seq2^29^. Briefly, following cell lysis, Poly(A)^+^ RNA was reverse transcribed using SuperScript II reverse transcriptase (Invitrogen) in a strand-switch reaction to add a reverse primer for the second-strand synthesis. The cDNA was amplified by PCR (18 cycles) using KAPA HiFi Hot-Start ReadyMix (KAPA Biosystems) and purified using Ampure XP beads (Beckman Coulter, California, USA). The quantity and quality of the cDNA libraries were assessed using an Agilent 2100 BioAnalyzer (Agilent Technologies, California, USA). For gene expression analysis, quantitative PCR reactions were carried out in a Roche LightCycler 480 II System (Roche, Basal, Switzerland) using FastStart Universal SYBR Green Master (Roche) under the following conditions: 95 °C for 5 min, followed by 45 cycles of denaturation at 95 °C for 15 s, annealing at 58 °C for 30 s, and extension at 72 °C for 30 s. We used the glyceraldehyde-3-phosphate dehydrogenase (*GAPDH*) gene as the internal standard, and calculated the average comparative threshold cycle (DCt) values for analysis. Supplementary Table S1 lists the primers used.

To evaluate the expression of pluripotency-associated genes, total RNA was extracted with TRIzol (Invitrogen) and cDNA was synthesized by RevertAid™ first strand cDNA synthesis kit (Fermentas Life Sciences, Burlington, Canada), according to the manufacturer’s instructions. A semiquantitative PCR reaction was carried out under the following conditions: 95 °C for 5 minutes; 30 cycles of amplification (95 °C for 30 seconds, 54~60 °C for 30 seconds and 72 °C for 30 seconds); and a final extension at 72 °C for 5 minutes. Supplementary Table S1 lists the primers used.

### Human parthenogenetic embryonic stem cells (pESCs): derivation and characterization

HPs were cultured to the blastocyst stage in G2 Plus media. Isolation of the inner cell mass (ICM) from a blastocyst was performed as previously described^30^. ICMs were cultured on mitotically inactivated mouse embryonic fibroblasts (mEFs) in DFSR medium, containing DMEM/F12 supplemented with 15% knockout serum replacement, 2 mM nonessential amino acids, 2 mM L-glutamine, 0.1 mM β-mercaptoethanol and 4ng/mL of basic Fibroblast Growth Factor (bFGF) (all from Invitrogen). The medium was changed every day and pESCs were passaged mechanically every 7 days. For the *in vitro* differentiation assay, pESCs colonies were dissociated mechanically into small clumps and detached to grow as aggregates in suspension for 14 days to form embryoid bodies (EBs) in DFSR medium without bFGF. The EBs were then transferred to matrigel-coated 6-well plates for adherent culture for 6 days in the same medium, followed by immunocytochemical analysis.

Total genomic DNA was extracted from the pESCs using a DNeasy Tissue kit (Qiagen, Venlo, Netherlands) and single nucleotide polymorphism (SNP) analysis was performed using a Genome-Wide Human SNP Array 5.0 (Affymetrix, California, USA). Allele difference was analysed using the Chromosome Analysis Suite (ChAS) 3.0. Moreover, pESC clones were transferred onto matrigel-coated dishes for propagation and prevention of mEFs contamination. After 2–3 passages, cells were treated with Demecolcine (100 ng/mL, Sigma) for 2.5 h and then dissociated into singe cells with Trypsin/ EDTA (0.05%, Invitrogen) for standard G-banding karyotype analysis. The analysis was based on 50 metaphase cells per sample.

### Diploidization by electrofusion two-cell stage HPs

HPs were picked up at the two-cell stage and assigned randomly into two groups: the electrofusion group and the non-fusion group (control). Diploid embryos were produced by fusion of blastomeres at the two-cell stage, according to the technique described by Cheong *et al*.^31^, with slight modifications, using a CF-150B pulse generator (BLS, Hungary) and an electrode-chamber with a 250-μm gap. In general, 30–34 h after activation, the cleavage plane of the two-cell embryos was first oriented in parallel with the electrodes by the application of 2 V AC, and then fusion of the cell membrane was induced by two 40 μs pulses of 30 v direct current. Embryos were removed from the fusion chamber quickly, washed with G-MOPS medium and cultured in G1 Plus media at 37.5 °C, 6% CO_2_, 5% O_2_, and 89% N_2_ in a humidified atmosphere. About 45 minutes later, the embryos were examined for complete fusion of the two blastomeres. In the non-fusion group, HPs were cultured directly in G1 Plus media. All embryos were cultured to the blastocyst stage and the culture medium was changed to G2 Plus media at day 3. The developmental potential of the embryos was evaluated between the fusion group and the non-fusion group.

### Statistical analysis

Statistical analysis was performed using the Statistical Package for Social Sciences (SPSS, version 19.0). Categorical variables were analysed by a chi-squared test or Fisher’s exact test and continuous variables were analysed by a t-test if they followed a normal distribution. Data that were not normally distributed were tested with a Mann-Whitney-Wilcoxon test. A P-value < 0.05 was considered statistically significant.

## Results

### Cleavage behaviour of haploid parthenotes

**Figure 1:**
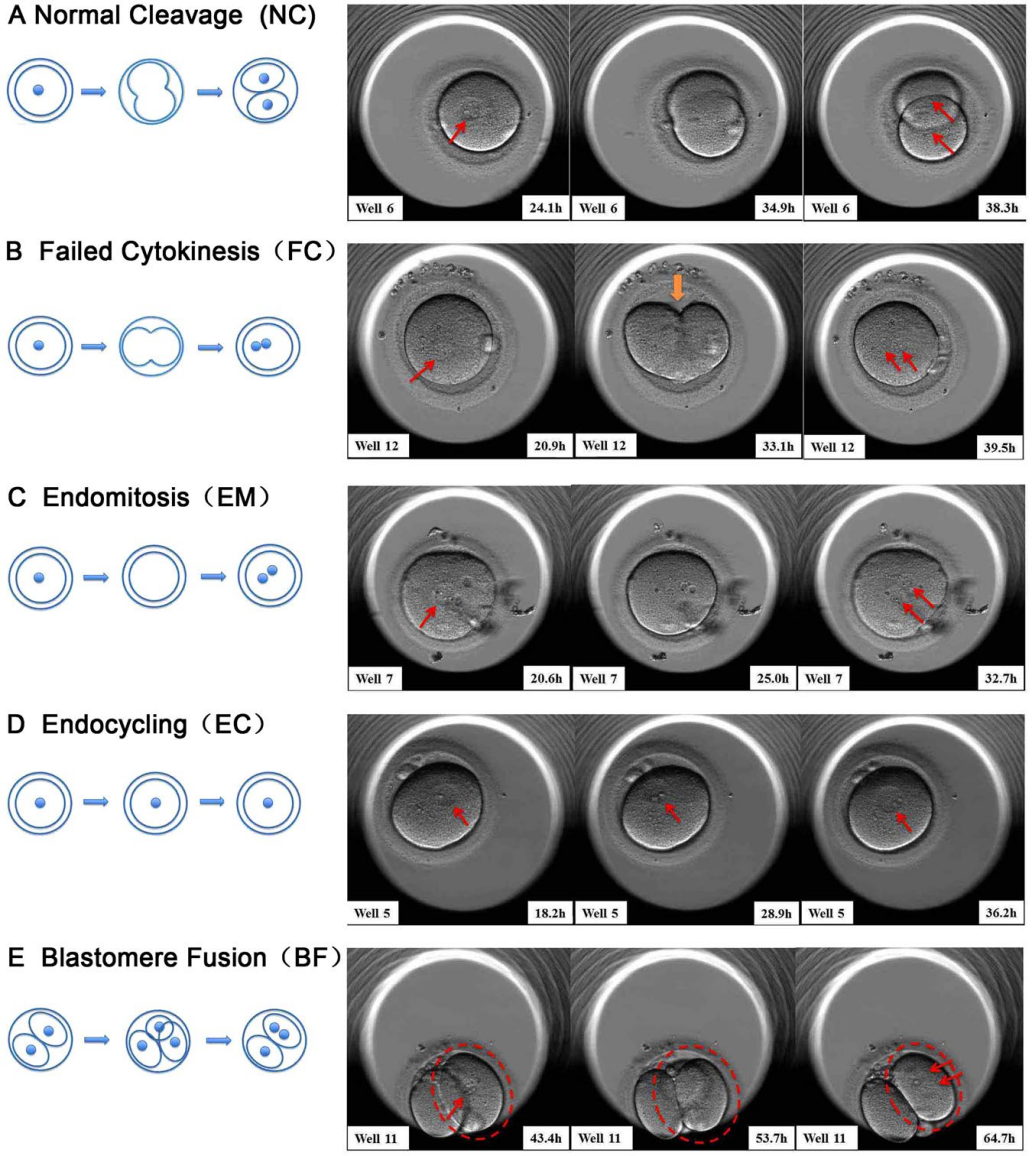
Normal and four kinds of self-diploidization-related cleavage behaviours were recorded by time-lapse photography in haploid parthenotes (HPs). (**A**) Normal cleavage (NC), cleavage from one cell to two even blastomeres; (**B**) Failed cytokinesis (FC), a cleavage furrow appeared but the cell recovered into one cell again rather than divide into two daughter blastomeres; (**C**) Endomitosis (EM), replication of the nucleus was complete, but there was no appearance of the cleavage furrow; (**D**) Endocycling (EC), the duration of the pronuclear phase was extended obviously and more than one cell cycle occurred; and (**E**) Blastomere fusion (BF), one cell divided into two daughter blastomeres, which fuse into one blastomere again after a mitotic cycle. The nucleus is indicated by a red arrow.

**Figure 2:**
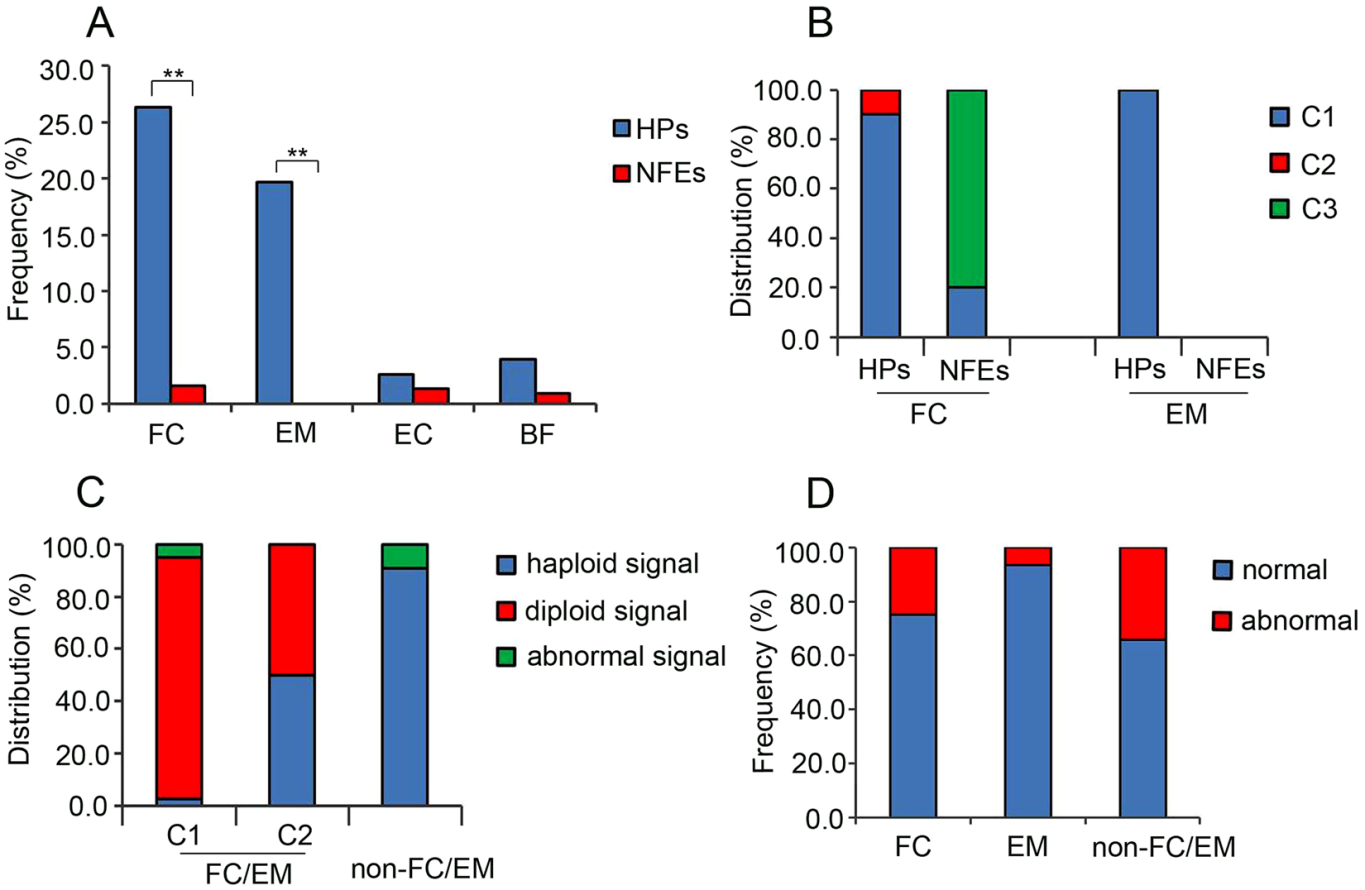
Characterization of FC/EM cleavage behaviours in HPs. (**A**) The frequency of FC/EM/EC/BF cleavage behaviours between HPs and NFEs; (**B**) The distribution of FC/EM during the first three cleavages between HPs and NFEs; (**C**) The percentage of haploid, diploid and abnormal signals appearing in FC/EM and non-FC/EM HPs; and (**D**) The percentage of normal and abnormal cleavages appearing in the next cell cycle between FC/EM and non-FC/EM HPs. HPs: haploid parthenotes, NFEs: normal fertilized embryos, FC: failed cytokinesis, EM: endomitosis, EC: endocycling, BF: blastomere fusion, C1: the first cell cycle, C2: the second cell cycle, C3: the third cell cycle. An asterisk indicates a significant difference by a t-test (P < 0.01).

A total of 242 *in vitro* matured metaphase (MII) oocytes were collected. Among them, 172 of the 216 activated oocytes extruded the second polar body and had one pronucleus; 11 of the 14 activated oocytes lacked the second polar body and displayed one pronucleus; and 10 of the 12 fertilized oocytes exhibited two pronuclei after an additional 16–18 h of culture. Under subsequent time-lapse monitoring, we observed four kinds of cleavage behaviours related to self-diploidization in HPs during the three initial divisions, which were defined as: (i) failed cytokinesis (FC, the cleavage furrow appeared but the cell recovered into one cell again rather than dividing into two daughter blastomeres) (Fig. 1B, Supplementary Video S2); (ii) endomitosis (EM, replication of the nucleus was complete; however, cytokinesis failed to initiate) (Fig. 1C, Supplementary Video S3); (iii) endocycling (EC, the duration of the pronuclear phase was obviously extended and more than one cell cycle occurred) (Fig. 1D, Supplementary Video S4); and (iv) blastomere fusion (BF, one cell divided into two daughter blastomeres, which then fused into one blastomere again after a mitotic cycle) (Fig. 1E, Supplementary Video S5). To clarify the incidence of FC/EM/EC/BF during preimplantation development, the time-lapse records of the HPs were analysed and compared with previously described data from 328 human NFEs^20^. As shown in Fig. 2A, the percentages of FC and EM were significantly higher in HPs than in NFEs (26.3% *vs*. 1.6%, P < 0.01; 19.7% *vs*. 0%, P < 0.01, respectively), while the frequencies of EC and BF did not differ significantly between HPs and NFEs (2.6% *vs*. 1.3%, P > 0.05; 3.9% *vs*. 0.9%, P > 0.05, respectively).

In 46.0% (35/76) of the HPs, FC/EM appeared during the first three cleavages. For FC, 90% (18/20) occurred in HPs at the first cell cycle and 10% (2/20) appeared at the second cell cycle. For NFEs, 20% (1/5) occurred at the first cleavage and 80% (4/5) appeared at the third cleavage. For EM, 100% (15/15) occurred in HPs at the first cell cycle and no NFEs appeared during the first three cleavages (Fig. 2B). To test whether diploidy was recovered in HPs after FC/EM behaviours, we performed FISH analysis using DNA probes specific for chromosomes 16, 18, and X. Twenty-four day-3 HPs were examined: 18 had signals and six failed during the fixation. When FC/EM appeared in the first cell cycle, the frequencies of haploid/diploid/abnormal signals were 2.6%, 92.1%, and 5.2%, respectively. When FC/EM occurred at the second cleavage, the frequencies of haploid/diploid/abnormal signals were 50%, 50%, and 0%, respectively. By contrast, the percentages of haploid/diploid/abnormal signals in non-FC/EM HPs were 90.9%, 0%, and 9.1%, respectively (Fig. 2C, Supplementary Table SII). Taken together, these results suggested that FC/EM behaviours in the first cleavage were associated highly with the self-diploidization of HPs.

We next sought to analyse the division behaviours in the following cell cycle after FC/EM occurred. We observed that 75.0% of FC HPs and 93.3% of EM HPs divided normally; however, 25.0% of FC HPs and 6.7% of EM HPs showed abnormal cleavage behaviours, including direct cleavage and fragmentation. The rate of normal division behaviours in the next cell cycle was higher in FC/EM HPs than in non-FC/EM HPs, but no significant difference was detected (75.0%, 93.3% *vs*. 65.8%, P > 0.05) (Fig. 2D). These observations revealed that FC/EM HPs might have a higher chance to progress normally during subsequent development.

### FC/EM improve the developmental potential of HPs

**Figure 3:**
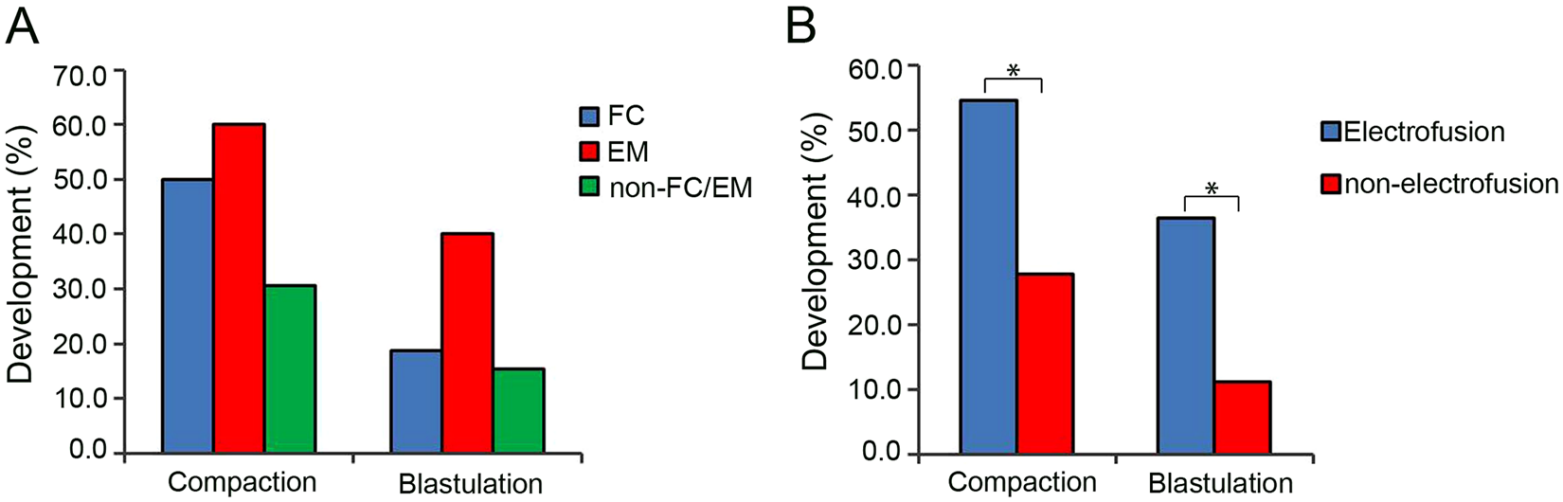
Diploidization could improve the developmental potential of HPs. (**A**) The percentage of compaction and blastulation of FC/EM and non-FC/EM HPs; (**B**) The percentage of compaction and blastulation of electrofusion and non-electrofusion HPs. HPs: haploid parthenotes, FC: failed cytokinesis, EM: endomitosis. An asterisk indicates a significant difference by a t-test (P < 0.05).

To investigate whether FC/EM behaviours could enhance the developmental potential of HPs, 16 FC, 10 EM, and 26 non-FC/EM HPs were cultured to day 6 to evaluate their ability to form blastocysts. We found that the percentages of FC/EM embryos that developed to the morula stage were higher than that of non-FC/EM embryos (50.0%, 60.0% *vs*. 30.7%, P > 0.05) (Fig. 3A). Similarly, higher rates of blastocysts were observed in the embryos of FC/EM compared with the non-FC/EM embryos (18.8%, 40.0% *vs*. 15.4%, P > 0.05) (Fig. 3A), suggesting that self-diploidization through FC/EM behaviours contributed to restoring the developmental ability of HPs.

**Figure 4:**
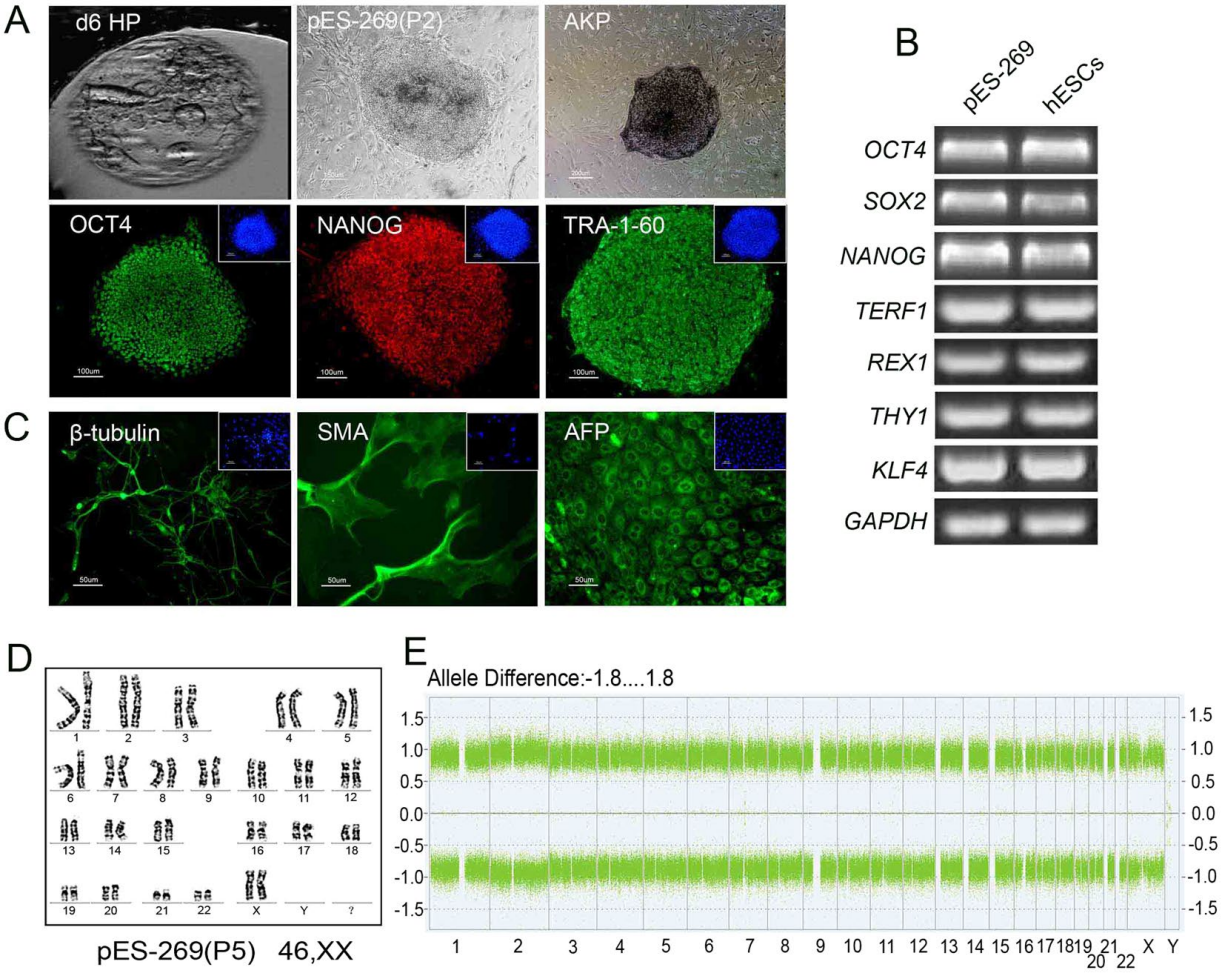
Isolation and characterization of pES-269 cells with commonly used markers. (**A**) pES-269 cells isolation, Alkaline phosphatase (AKP) staining and immunofluorescence staining using anti-OCT4, -NANOG and -TRA-1-60 antibodies; (**B**) RT-PCR assays for the expression of pluripotency-associated markers in pES-269 cells; (**C**) Immunostaining of differentiated cells from embryoid bodies (EBs) formed by pES-269 cells, using antibodies against β-tubulin (ectoderm), SMA (mesoderm), and AFP (endoderm); (**D**) Karyotype analysis of pES-269 cells; (**E**) Genome-wide SNP analysis of pES-269 cells using an Affymetrix 250 K SNP Array. A homozygous AA allele maps to approximately +1, and a homozygous BB allele maps to approximately −1, with the heterozygote mapping to approximately 0.

To further investigate the developmental potential of blastocysts from self-diploidization, eleven blastocysts were used to derive ESC lines. One stable pESC line (pES-269) was established successfully from a blastocyst, which occurred via FC at the first cleavage (Fig. 4A). The pES-269 line exhibited a normal diploid karyotype of 46, XX (Fig. 4D), and displayed typical features of hESCs, including alkaline phosphatase-positivity and expression of hESC-specific markers OCT4, NANOG, and TRA-1-60 (Fig. 4A). RT-PCR assays suggested that pES-269 expressed pluripotency related genes strongly, including *OCT4*, *NANOG*, *SOX2*, *REX1*, *THY1*, *KLF4* and *TERF1* (Fig. 4B). The developmental potential of pES-269 cells was evaluated via *in vitro* differentiation. EBs formed when pES-269 cells were cultured in suspension and various types of cells grew out of the EBs after attachment. Immunocytochemical analysis revealed the presence of cells expressing ectoderm (β-tubulin), mesoderm (SMA), and endoderm (AFP) markers (Fig. 4C). EB differentiation demonstrated that pES-269 possessed the potential to develop into differentiated derivatives of all three primary germ layers. When we examined the genomic homozygosity of pES-269 using a genome-wide SNP microarray, 98.9% of markers from autosomes and the X chromosome were homozygous (Fig. 4E), indicating that the genome of pES-269 came from duplication of a haploid genome.

### Diploidization via electrofusion could improve blastocyst formation effectively in HPs

FC/EM behaviours are likely to occur at the first cell cycle and could enhance the developmental ability of HPs. Therefore, we next examined whether artificial diploidization at the first cell cycle could improve the developmental potential of HPs. Two-cell stage HPs were divided into two groups: electrofusion and non-electrofusion. In the electrofusion group, we observed that 54.5% (12/22) of embryos were compacted and 36.4% (8/22) of embryos developed to the blastocyst stage, whereas the percentages of compaction and blastocyst formation of the non-electrofusion group were 27.8% (5/18) and 11.1% (2/18), which were significantly lower than those of the electrofusion group (P < 0.05) (Fig. 3B).

### M phase progression in FC/EM embryos in the first cleavage

**Figure 5:**
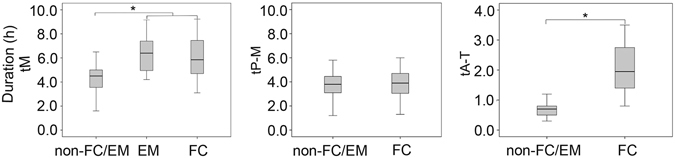
The duration of M phase of the 1st cell cycle between FC/EM and non-FC/EM HPs. HPs: haploid parthenotes, FC: failed cytokinesis, EM: endomitosis; tM: the duration of the disappearance of the pronucleus to the reappearance of the nucleus; tP-M: the duration of the disappearance of the pronucleus to the initiation of cytokinesis; tA-T: the duration of the initiation of cytokinesis to complete cytokinesis and the reappearance of the nucleus. An asterisk indicates a significant difference by a t-test (P < 0.05).

To gain an insight of the mechanism of self-diploidization, we further characterised the M phase kinetics in the first cleavage of HPs. We investigated the duration of the M phase (tM, the duration of the disappearance of the pronucleus to the reappearance of the nucleus) of the 1st cell cycle between FC/EM and non-FC/EM HPs. As shown in Fig. 5, FC/EM HPs took longer than non-FC/EM (tM, 5.9 (4.6–7.5) h, 6.4 (4.9–7.7) h *vs*. 4.5 (3.4–5.0) h, P < 0.05, respectively). We then analysed which phase was responsible for the extension of the M phase. The duration of prophase-metaphase (tP-M, the duration of the disappearance of the pronucleus to the initiation of cytokinesis) and anaphase-telophase (tA-T, the duration of the initiation of cytokinesis to complete cytokinesis and the reappearance of the nucleus) were analysed. There was no difference in the length of the tP-M between FC and non-FC/EM HPs (3.9 (3.0–4.8) h *vs*. 3.8 (2.9–4.5) h, P > 0.05). However, the tA-T was significantly longer in FC compared with non-FC/EM HPs (1.95 (1.4–2.8) h *vs*. 0.7 (0.5–0.9) h, P < 0.01) (Fig. 5). Thus, the extension of cytokinesis was the main cause of M phase elongation.

### Compromised RhoA pathway activation in the M phase of HPs

**Figure 6:**
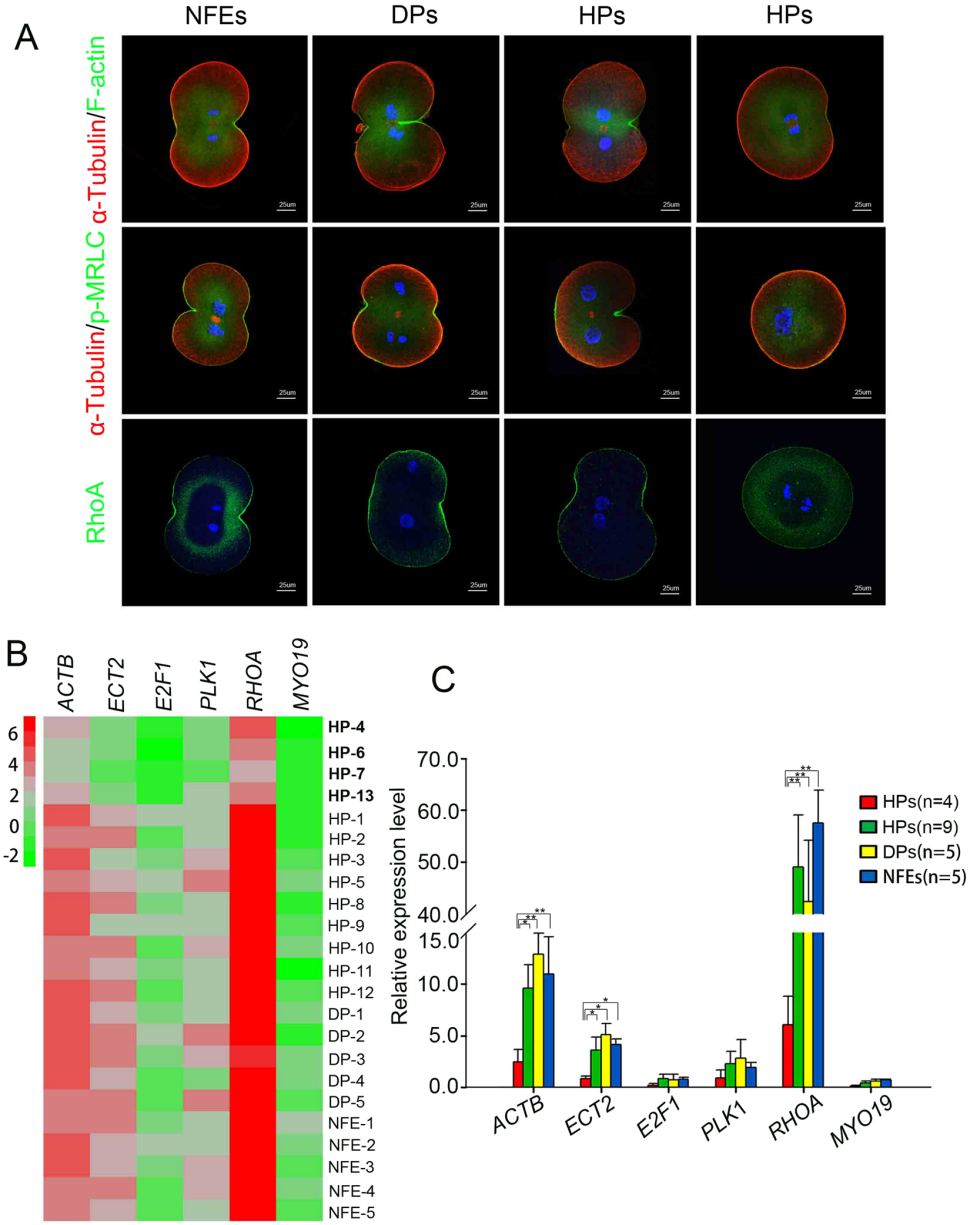
Self-diploidization of HPs might be result from a defect in Rho pathway activation. (**A**) Immunostaining of NFEs, DPs, and HPs at the first division stage, using antibodies against F-actin, p-MRLC, RhoA, and α-Tubulin; (**B**) Heatmap showing the relative expressions of the six core components of the Rho pathway detected by quantitative RT-PCR; (**C**) Quantitative RT-PCR analysis of the expression levels of the Rho pathway genes. An asterisk indicates a significant difference by a t-test (P < 0.05). NFEs: normal fertilized embryos, DPs: diploid parthenotes, HPs: haploid parthenotes, p-MRLC: serine19-phosphorylated myosin regulated light chain.

The RhoA pathway is a central player in the assembly of the contractile ring during cytokinesis. Activated RhoA regulates actin polymerization and myosin activation at the midzone via interactions with different effectors^32^. Therefore, we investigated the expression and localization of activated RhoA in HPs at the first cleavage stage. Using the TCA fixation method, we found that RhoA was highly concentrated in the midzone cortex in all NFEs and DPs, and in 33.3% (2/6) of HPs. Nevertheless, 66.7% (4/6) of the HPs remained round and did not exhibit furrow ingression after the pronucleus disappeared for several hours (3–5 h). These HPs showed no evident accumulation of RhoA. We further examined other principal components of the contractile ring; actin filaments (F-actin) and serine19-phosphorylated myosin regulate light chain (p-MRLC). As shown in Fig. 6A, an accumulation of F-actin, appearing as a band of fluorescence, was clearly observed at the site of furrow ingression in all NFEs and DPs, and in 60% (3/5) of HPs, while 40% (2/5) of the HPs showed incomplete concentration of F-actin around the midzone. For p-MRLC, all NFEs and DPs, and 40% (2/5) of HPs showed an obvious accumulation in the midzone, whereas 60% (3/5) of the HPs did not show any detectable accumulation on the cell cortex. Moreover, we examined the distribution of α-Tubulin in the central spindle. We observed that α-Tubulin was located appropriately in NFEs, DPs, and normally divided HPs. Among the failed cytokinesis HPs, only one embryo showed a clear localization in the central spindle. In addition, we observed that failed cytokinesis HPs presented varied nuclear masses, including a single nucleus, two separated nuclei and two side-by-side nuclei. However, most of the HPs failed to form two sets of completely separated nuclear masses (Fig. 6A). These observations suggested that defects in the expressions of RhoA, F-actin, and p-MRLC might be responsible for the failure of cytokinesis in HPs.

To gain an additional insight into the mechanism of self-diploidization, we assessed the expressions of core components in the RhoA pathway, including *ACTB*, *ECT2*, *E2F1*, *PLK1*, *RHOA* and *MYO19*. We collected five NFEs, five DPs, and 13 HPs for gene expression analysis. There was no significant difference between the NFEs and DPs in the expression of the six genes. Among the HPs, 69.2% (9/13) of the embryos showed similar levels compared with the NFEs or DPs, whereas 30.8% (4/13) of the embryos displayed reduced expression of *ACTB*, *ECT2* and *RHOA* (P < 0.05) (Fig. 6B). These results indicated that four of the HPs had an impaired Rho pathway activity and were thus more likely to experience failure of cytokinesis during cell division.

## Discussion

Recent reports have shown that haploid parthenogenetic and/or androgenetic ESCs exhibit an intrinsic tendency for diploidization following passaging *in vitro* and diploidization greatly hinders the application of haploid ESCs for loss-of-function screens. However, the exact mechanism responsible for diploidization remains to be determined. Previous studies had revealed the relationship between increased ploidy and abnormal cleavage behaviors of FC/EM/EC/BF in megakaryocytes and human embryos^18, 26, 33, 34^. In this study, although the above four types of cleavage behaviours were observed in HPs through time-lapse analysis, the frequency of EC/BF did not differ significantly between HPs and NFEs. It is possible that EC/BF are random abnormal behaviours during preimplantation development and could not account for the high frequency of self-diploidization. By contrast, we observed a significantly increased frequency of FC and EM in HPs compared with NFEs, and HPs exhibited a diploid signal after FC/EM occurred. These results indicated that the two behaviours might be highly correlated with the self-diploidization of HPs.

FC/EM cleavage behaviours are normal physiological behaviours during megakaryocytopoiesis^26^. In the process of self-diploidization, a behaviour that does not affect and improve the developmental potential of embryos would be the real mechanism responsible for self-correction of HPs. In our study, we observed that FC/EM did not influence the cleavage behaviour in the subsequent cell cycles of HPs, and a higher percentage of FC/EM HPs had a chance to progress across the 8-cell stage and develop to the blastocyst stage compared with non-FC/EM HPs. Furthermore, artificial diploidization improved blastocyst formation significantly in HPs. Some of these blastocysts were of good quality containing both TE and ICM parts, which was confirmed by later successful pluripotent stem cell derivation. Taken together, these results confirmed that recovery of diploidy is important to improve the developmental potential of HPs.

Previous studies indicated that failure of cytokinesis correlates with a partial defect in the activation of the Rho pathway. RhoA is concentrated in the midzone cortex during anaphase or at the cleavage furrow during telophase. The Rho pathway plays a crucial role in cytokinesis by activating actin polymerization and myosin II activity to form the contractile ring and later furrow ingression. In the present study, RhoA, F-actin, and p-MRLC signals were not detected by immunostaining in some HPs, which, together with the reduced expression of *ACTB*, *ECT2* and *RHOA* compared with NFEs or DPs, indicated that the defect in Rho pathway activation is responsible for the failure of cytokinesis. The E2Fs/ECT2 pathway, which regulates RhoA activation and is required for cytokinesis in mammalian cells^35, 36^, is a downstream target of RB-family-dependent transcriptional repression induced by DNA damage. We observed that 30.8% of HPs showed reduced expression of *ECT2* compared with NFEs or DPs. Thus, whether the low DNA content in HPs might result in some characteristics that are similar to DNA damage to downregulate the E2Fs/ECT2 pathway requires confirmation by further studies.

In conclusion, our study provided primary evidence that human HPs could achieve diploidization actively through Rho pathway-regulated FC/EM cleavage behaviours. Further fine tuning of this signalling pathway might help to either generate stable haploid embryonic stem cell lines for developmental biology studies, or improve the efficiency for diplodization to generate diploid homozygous embryonic stem cells, which have potential human leucocyte antigen (HLA)-match superiority in regenerative medicine.

## Electronic supplementary material

Video S1

Video S2

Video S3

Video S4

Video S5

supplemental materials
