# Supplementary material for: Self-diploidization of human haploid parthenogenetic embryos through the Rho pathway regulates endomitosis and failed cytokinesis
Source: Sci Rep. 2017 Jun 26;7:4242. doi: 10.1038/s41598-017-04602-y (PMC5484709; doi:10.1038/s41598-017-04602-y)
Supplement: Supplementary file 6 — supplemental materials [file 41598_2017_4602_MOESM6_ESM.pdf]

Self-diploidization of human haploid parthenogenetic embryos through the Rho pathway regulates endomitosis and failed cytokinesis

Lizhi Leng[1,2]*, Qi Ouyang[1,2,3]*, Xiangyi Kong[1,2]*, Fei Gong[1,2], Changfu Lu[1,2], Lei Zhao[1,2], Yun Shi[1,2], Dehua Chen[1,2], Liang Hu[1,2,3], Guangxiu Lu[1,2,3], Ge Lin[1,2,3]

1 Institute of Reproductive & Stem Cell Engineering, Central South University, Changsha 410078, China

2 Key Laboratory of Stem Cells and Reproductive Engineering, Ministry of Health, Changsha 410078, China

3 National Engineering and Research Center of Human Stem Cell, Changsha 410078, China

* These authors contributed equally to this work.

Correspondence should be addressed to G. Lin, Tel.: 86-731-84805319; Fax: 86-731-84497661; E-mail: linggf@hotmail.com

Supplemental Materials

Video legends

Video S1 Normal cleavage (NC), cleavage from one cell to two even blastomeres.

Video S2 Failed cytokinesis (FC), a cleavage furrow appeared but the cell recovered into one cell again rather than divide into two daughter blastomeres.

Video S3 Endomitosis (EM), replication of the nucleus was complete, but there was no appearance of the cleavage furrow.

Video S4 Endocycling (EC), the duration of the pronuclear phase was extended obviously and more than one cell cycle occurred.

Video S5 Blastomere fusion (BF), one cell divided into two daughter blastomeres, which fuse into one blastomere again after a mitotic cycle.

Supplementary Table SI. Primers used for RT-PCR and Quantitative RT-PCR**.**

Genes	Forward primers（5'-3'）	Reverse primers（5'-3'）	Tm (°C)
ACTB	ATGATGATATCGCCGCGCTC	CCACCATCACGCCCTGG	58
ECT2	AGTTGTCCTGGAAAATCGGATGA	TGGCAAAGGCTCTCCTTTTTG	59
E2F1	ACAAGGCCCGATCGATGTTT	AACAGCGGTTCTTGCTCCAG	58
PLK1	CCGCAATTACATGAGCGAGC	TGAGCTTGGTGTGATCCTGG	58
RHOA	AGCCAAGATGAAGCAGGAGC	TTCCCACGTCTAGCTTGCAG	56
MYO19	CAGAGAGGAGCTTAGAAGAGGA	CCAAACTTAAGTGGCGTGGC	58
SOX2	AGTCTCCAAGCGACGAAAAA	GCAAGAAGCCTCTCCTTGAA	54
NANOG	TGAACCTCAGCTACAAACAG	TGGTGGTAGGAAGAGTAAAG	64
TERF1	GCAACAGCGCAGAGGCTATTATT	AGGGCTGATTCCAAGGGTGTAA	58
OCT3/4	CTTGCTGCAGAAGTGGGTGGAGGAA	CTGCAGTGTGGGTTTCGGGCA	64
REX1	TGAAAGCCCACATCCTAACG	CAAGCTATCCTCCTGCTTTGG	58
THY1	AGAATACCAGCAGTTCACCCATCC	CCTCACACTTGACCAGTTTGTCTCTG	58
KLF4	TCTCAAGGCACACCTGCGAA	TAGTGCCTGGTCAGTTCATC	58
GAPDH	ACCACAGTCCATGCCATCAC	CCACCACCCTGTTGCTGTA	58

Supplementary Table SII. Fluorescence in-situ hybridization on human haploid parthenogenetic embryos.

Cleavage behaviour	No.	Number of blastomeres	Sign of normal haploid	Sign of normal diploid	Abnormal sign
I-FC	E10	8C	1R1G1A(1)	2R2G2A(7)	
I-FC	E14	7C		2R2G2A(7)	
I-FC	E6	6C		2R2G2A(5)	3R2G3A(1)
II-FC	E6	8C	1R1G1A(4)	2R2G2A(4)	
I-EM	E17	4C		2R2G2A(4)	
I-EM	E18	4C		2R2G2A(3)	3R2G2A(1)
I-EM	E19	4C		2R2G2A(4)	
I-EM	E3	5C		2R2G2A(5)	
non-FC/EM	E1	5C	1R1G1A(5)		
non-FC/EM	E3	8C	1R1G1A(6)		
non-FC/EM	E4	7C	1R1G1A(5)		
non-FC/EM	E5	7C	1R1G1A(7)		
non-FC/EM	E7	8C	1R1G1A(7)		5R4G4A(1)
non-FC/EM	E4	6C	1R1G1A(6)		
non-FC/EM	E3	7C	1R1G1A(5)		1R2G1A(1)<br>2R1G3A(1)
non-FC/EM	E11	6C	1R1G1A(6)		
non-FC/EM	E12	8C	1R1G1A(8)		
non-FC/EM	E2	8C	1R1G1A(5)		1R4G2A(1)<br>3R1G1A(1)<br>1R3G2A(1)
